# Vortex 4.0 on chip

**DOI:** 10.1038/s41377-020-00343-2

**Published:** 2020-06-16

**Authors:** Qing Zhang, Jincheng Ni, Cheng-Wei Qiu

**Affiliations:** Department of Electrical and Computer Engineering, National University of Singapore, Singapore, 117583 Singapore

**Keywords:** Optical physics, Micro-optics

## Abstract

The orbital angular momentum (OAM) of light has promising applications, ranging from information multiplexing andhigh-speed optical communication to computation. Dynamically tunable and switchable vortex microlasers combinedwith direct photocurrent detection of the topological charges of OAM states have paved unexplored routes for thedevelopment and integration of fourth-generation (4.0) vortex technology, potentially on chip.

Chiral light with angular momentum has demonstrated a new degree of freedom in light–matter interactions, which lies at the heart of both fundamental theoretical research and advanced applications in modern physics. The photonic spin angular momentum (SAM) associated with the circular polarization of light is limited by two states. In contrast, the orbital angular momentum (OAM) of a vortex beam with an azimuthal phase gradient creates a new dimension with unlimited states in principle, and it has been widely applied in the fields of optical manipulation^1^, imaging and microscopy^2^, and quantum information processing^3^. In particular, unbounded OAM states provide additional channels for data transmission in optical communications. Therefore, dynamically tunable vortex light emitters and detectors have become essential for creating these emerging photonic technologies based on the OAM degrees of freedom.

On the one hand, previous three generations of approaches for vortex light sources remain static and involve the use of bulky components, such as spiral phase plates^4^ and forked holograms^5^ with spatial light modulators (SLMs). Recently developed planar optical components, including various metasurfaces^6^ and chip-scale microlasers^7^, offer a much more compact and robust solution to obtain highly pure coherent vortex modes and have recently been investigated extensively. However, so far, the demonstrated miniaturized vortex lasers that operate at telecommunication wavelengths lack reconfigurability and are limited by their output of one predefined polarized OAM state per wavelength. On the other hand, to detect the OAM order of light beams, many devices, such as SLMs, metasurfaces and gratings, have also been proposed in many publications^8,9^. Nevertheless, most of these approaches are optically addressed, and hence, on-chip detection of different OAM orders through direct electrical readout is strongly demanded but still remains unexplored.

Currently, two papers^10,11^ have been published in *Science* side by side to address these challenges in OAM-based optical communication on chip. Zhang et al.^10^ reported an OAM-tunable vortex microlaser that provides chiral light states of variable topological charges at a single telecommunication wavelength. Ji et al.^11^ demonstrated an OAM photodetector based on tungsten ditelluride (WTe_2_) to facilitate direct characterization of the topological charge of a vortex beam. These two works provide routes for the development of fourth-generation (4.0) vortex technology on chip.

A combined schematic of the front-end vortex microlaser and the back-end photodetector is sketched in Fig. 1. The tunable microlaser consists of a primary microring cavity made of an InGaAsP quantum well, which is coupled to a nearby bus waveguide with two control arms. The microring cavity supports two degenerate clockwise (↻) and counterclockwise (↺) whispering gallery modes. Due to the spin-orbit interaction, the two modes are locked to the right-hand (↓) and left-hand (↑) circular polarizations, represented by |*l*, ↻, ↓〉 and |*l*, ↺, ↑〉, respectively. Strategically, the authors exploited an external pumping at one of the bus waveguide arms to create gain (generated through pumping) and loss (intrinsic material loss without pumping) contrast and thus enabled a non-Hermitian interaction between the two circulating modes in the microring. When the left (or right) control arm is selectively pumped, unidirectional emission of |*l*, ↻, ↓〉 (or |*l*, ↺, ↑〉 vortex light is achieved. This non-Hermitian symmetry breaking provides a valuable method of vortex laser generation with controllable chirality. Moreover, the authors used an additional radial polarizer to further tune the OAM charge of the emitted vortex light. The radial polarizer converts circularly polarized (*σ* = ±1) light to a linearly polarized (*σ* = 0) beam but preserves the rotational symmetry of the emitted laser beam; the total angular momentum, *J* = *l* + *σ*, of light must remain conserved. Thus, for a given *J* = 2, the generated vortex beam has *l* = ±1 and *σ* = ±1 in the absence of a radial polarizer; when the laser beam passes through the polarizer, new OAM states with *l* = ±2 are achieved after the transfer of *σ* = ±1 SAMs to *l* = ±1 OAMs. The ability to simultaneously and cohesively manipulate both the SAM and OAM degrees of freedom offers fundamentally new functionality for controllable vortex light emission in a scalable way.

**Fig. 1:**
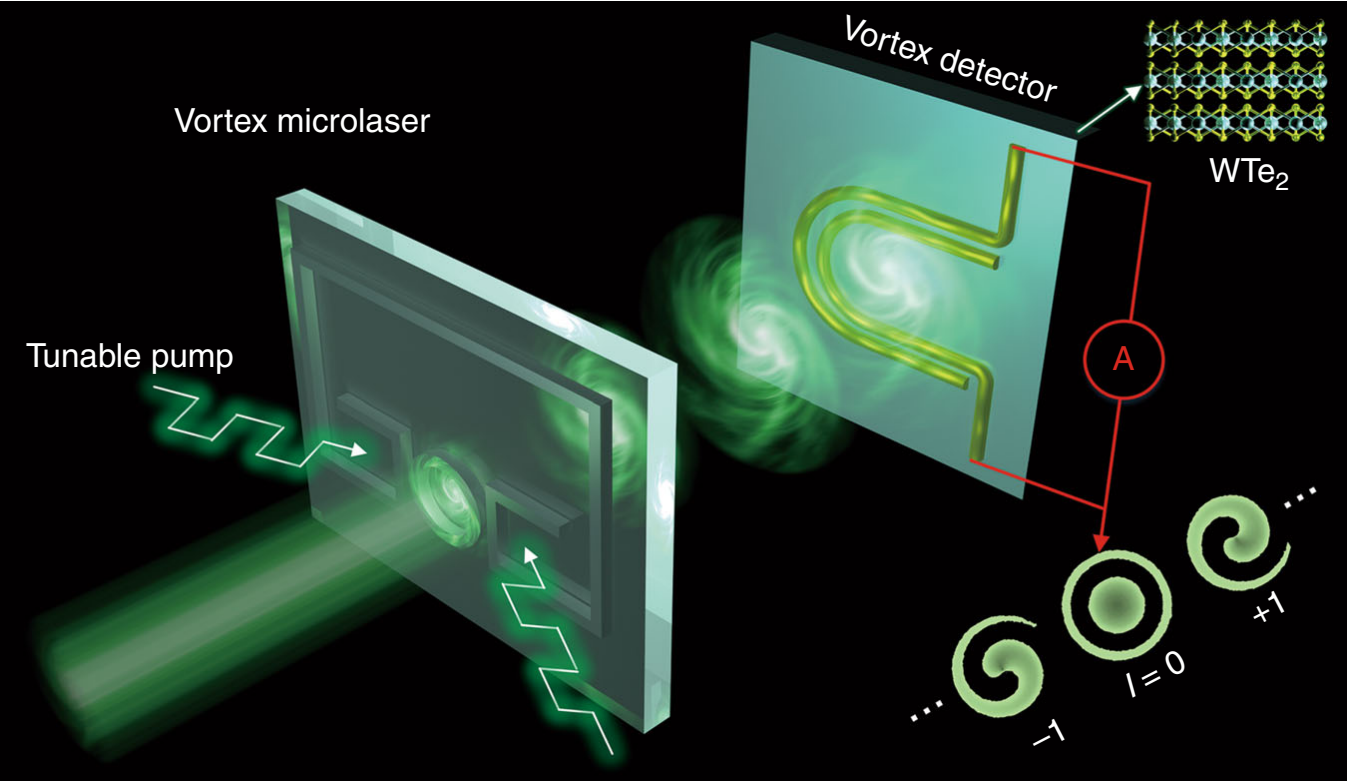
Schematic of a non-Hermitian controlled vortex microlaser^10^ and photocurrent detection of the orbital angular momentum (OAM) states of the generated vortex light^11^. The non-Hermitian interaction mediated by the externally applied control pump on the bus waveguide can be flexibly switched for the emission of OAM states with desirable chirality from the spin–orbit engineered microring. The vortex beam emitted on the WTe_2_ photodetector induced a nonlocal photocurrent with a magnitude proportional to its quantized OAM order. Hence, the measurement of photocurrent amplitudes can directly read out the topological charges of the OAM of light

To identify the OAM states at the back end, Ji et al. first reported an electrical approach for direct photocurrent detection of the OAM of light. The photodetector is made of multilayered two-dimensional WTe_2_ material with two U-shaped electrodes on top of the WTe_2_ surface (Fig. 1). When the OAM state *l* is nonzero, in addition to the linear momentum (*k*_0_*ħ*), an azimuthal photon momentum (*lħ*) results from the helical phase. Hence, a second-order photogalvanic conductivity tensor *β*_*ijkl*_ can be expanded to the first-order *α*_*ijkl*_ to obtain the DC current response, where the subscripts *i*, *j*, *k*, and *l* denote directions in the cylindrical coordinate system. The first-order *α*_*ijk*_ leads to a conventional local current response, which is related to the intensity and polarization of light, but it loses the helical phase information of OAM light. However, the second-order *β*_*ijkl*_ goes beyond the dipole approximation and describes a part of the spatially nonlocal current that is proportional to the quantized OAM order. This nonlocal current is termed the orbital photogalvanic effect current (*J*_OPGE_), and it can be divided into linear polarization-dependent parts (*J*_*L*_) and circular polarization-dependent parts (*J*_*c*_). The experimental results show that the circular part *J*_*c*_ displays step-like changes from OAM order −4 to 4. When the OAM index reverses the sign, the direction of the *J*_*c*_ current also flips. Hence, the measurement of the *J*_*c*_ amplitudes can directly indicate the topological charges of the OAM of light. Furthermore, the authors also indicated that for arbitrary vectorial OAM beams with space-variant states of polarization, both the OAM states and the polarization can be specifically determined by measuring currents via a small matrix of paired electrodes.

These techniques will find applications in on-chip optical communication, computing, and information security in both classical and quantum regimes. Dynamic switching between different OAM modes over time not only enables high-density data transmission but also increases the security of wired and wireless communication networks, and it may find expanded applications in optical tweezers, high-order quantum entanglement, high-capacity holographic encryption and nonlinear optics. In addition, direct reading of OAM states through photocurrent signals is possible, thus potentially facilitating the development of next-generation OAM-based photonic circuits. More effort and progress can be anticipated for integrated OAM transceiver modules in the near future.
